# Supplementary figures and images for: Prioritizing areas for post-fire restoration in Greece using mixed-methods spatial analysis
Source: PLoS One. 2026 Jan 12;21(1):e0339998. doi: 10.1371/journal.pone.0339998 (PMC12795358; doi:10.1371/journal.pone.0339998)

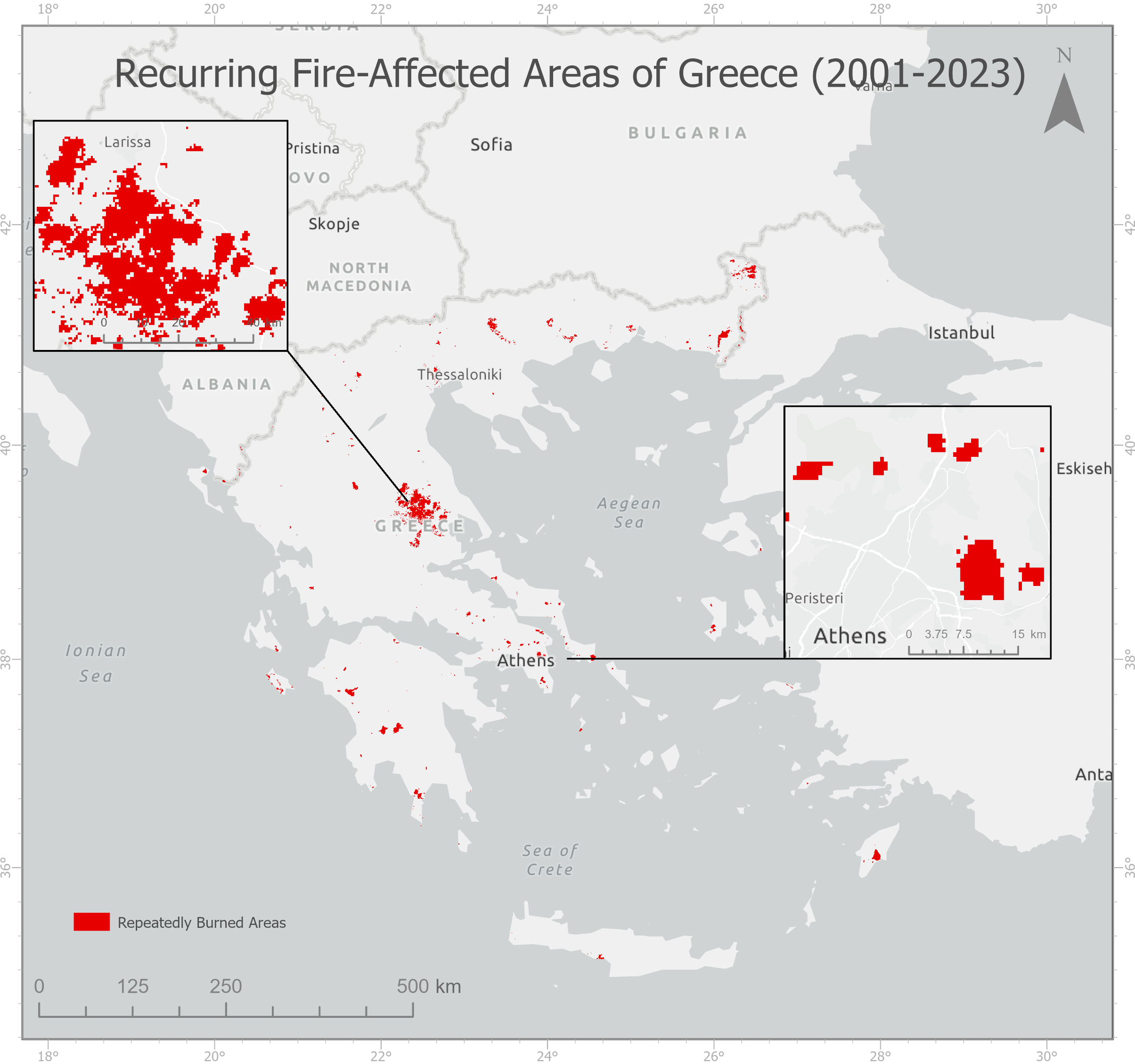

Supplement: S1 Fig — (TIF) [file pone.0339998.s001.tif]

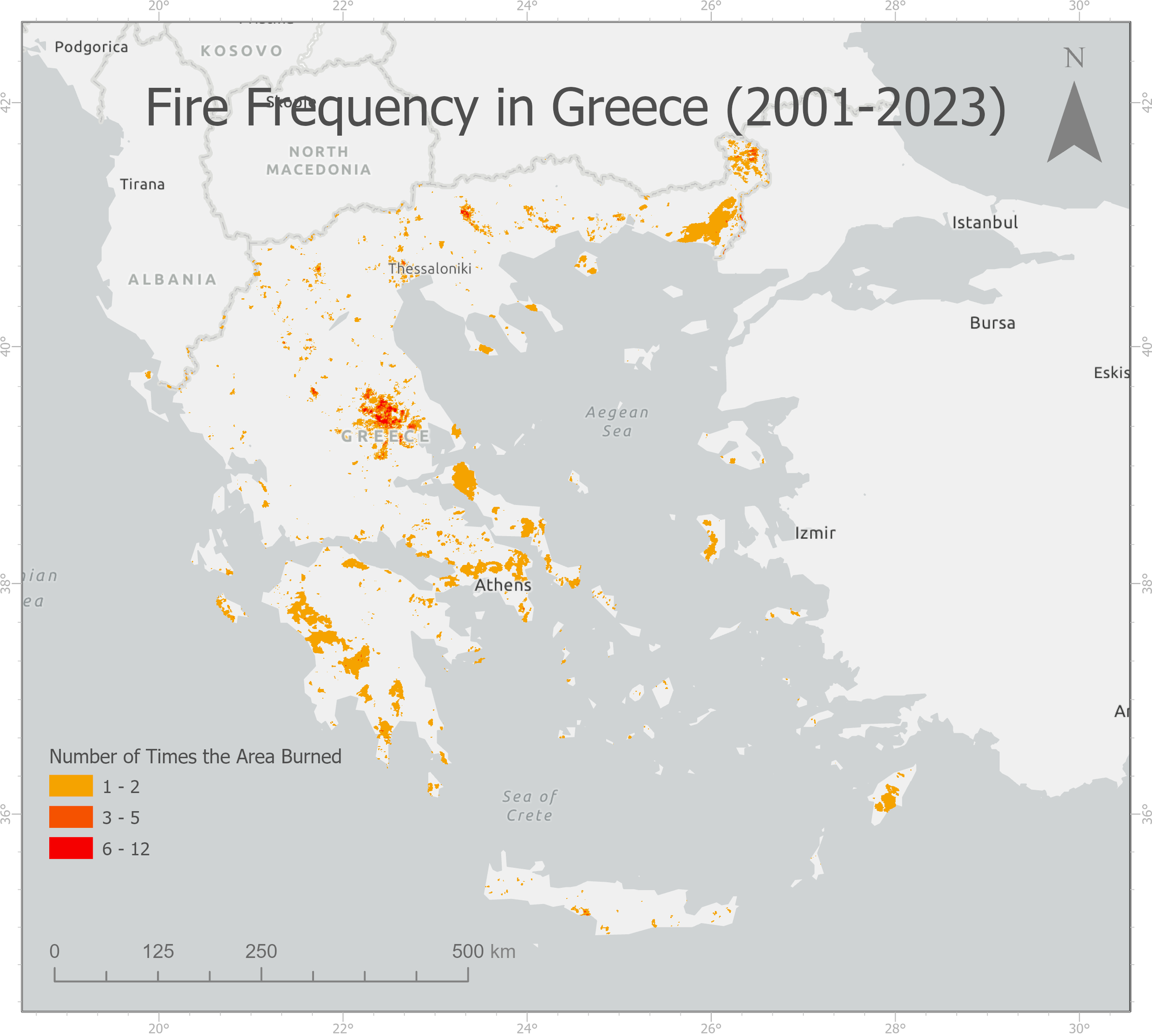

Supplement: S2 Fig — (TIF) [file pone.0339998.s002.tif]

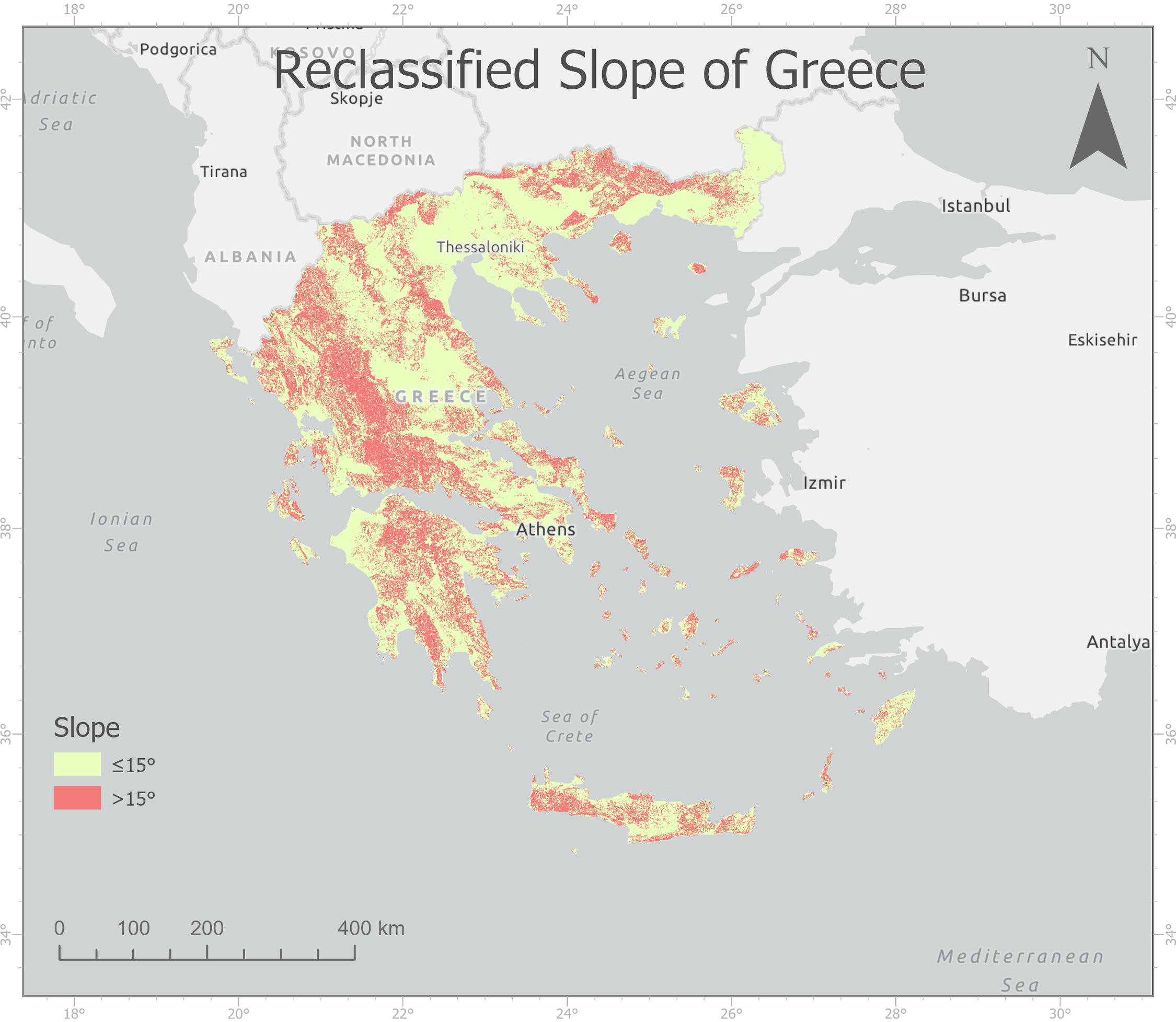

Supplement: S3 Fig — (TIF) [file pone.0339998.s003.tif]

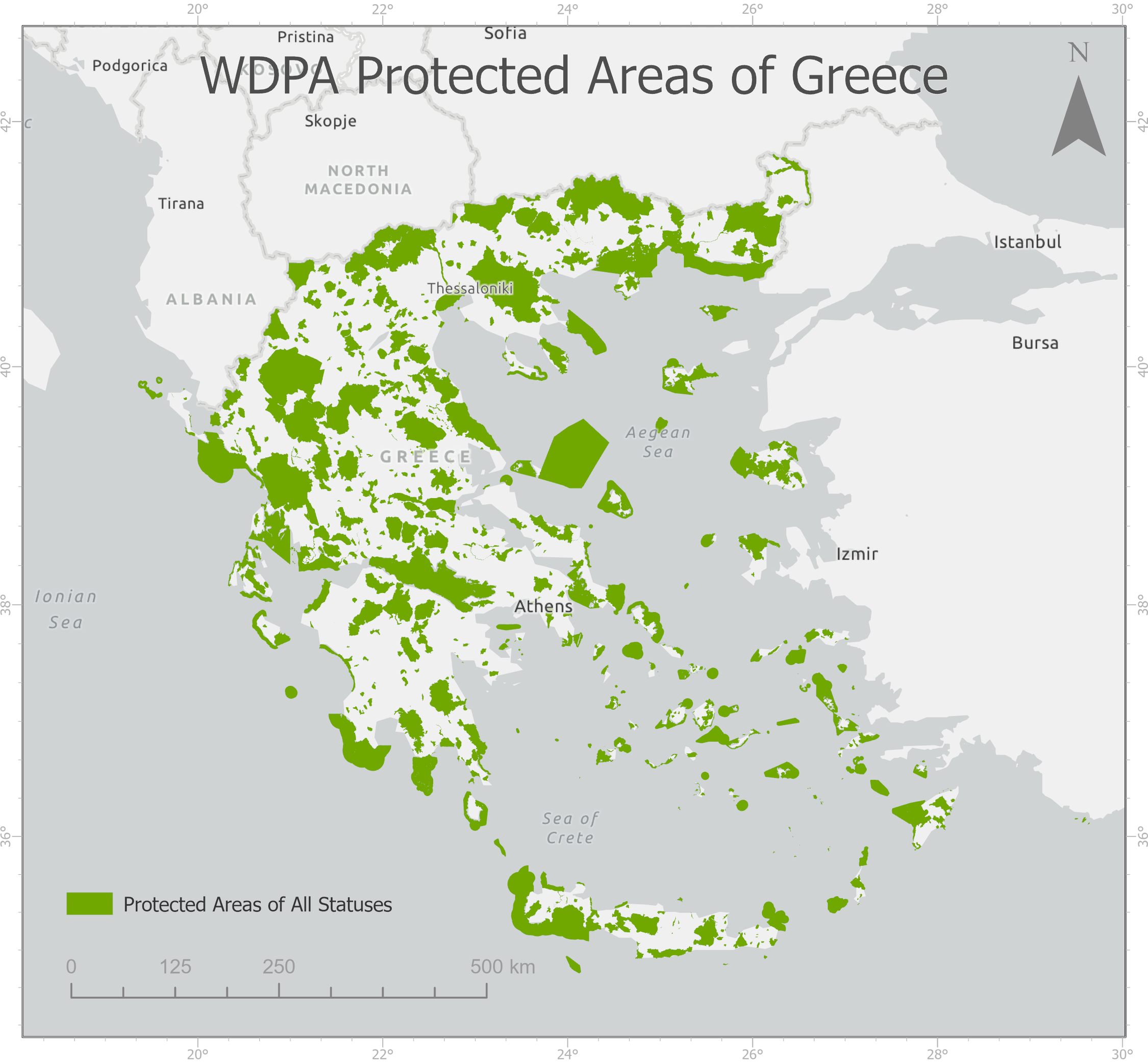

Supplement: S4 Fig — (TIF) [file pone.0339998.s004.tif]

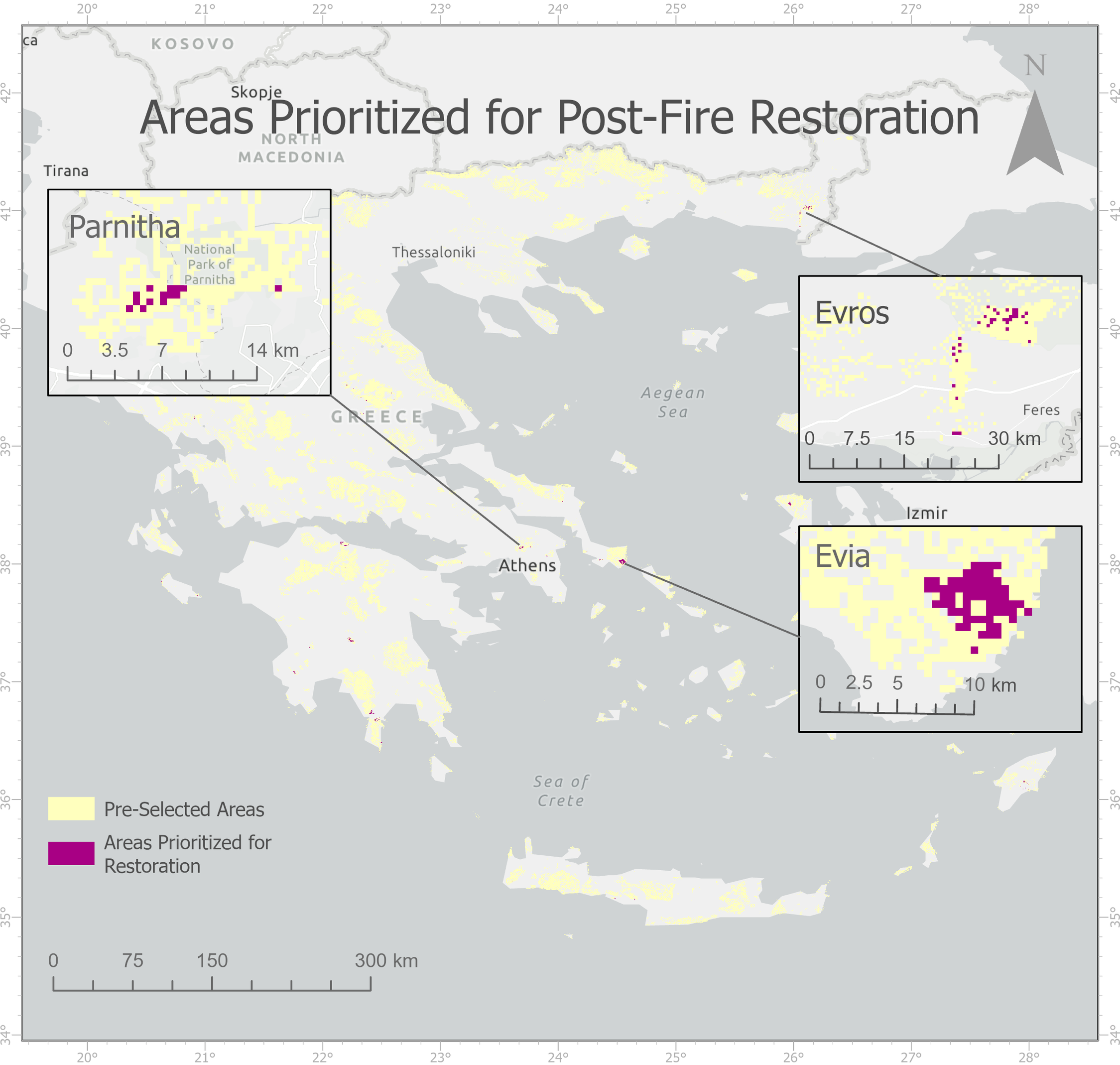

Supplement: S5 Fig — (TIF) [file pone.0339998.s005.tif]

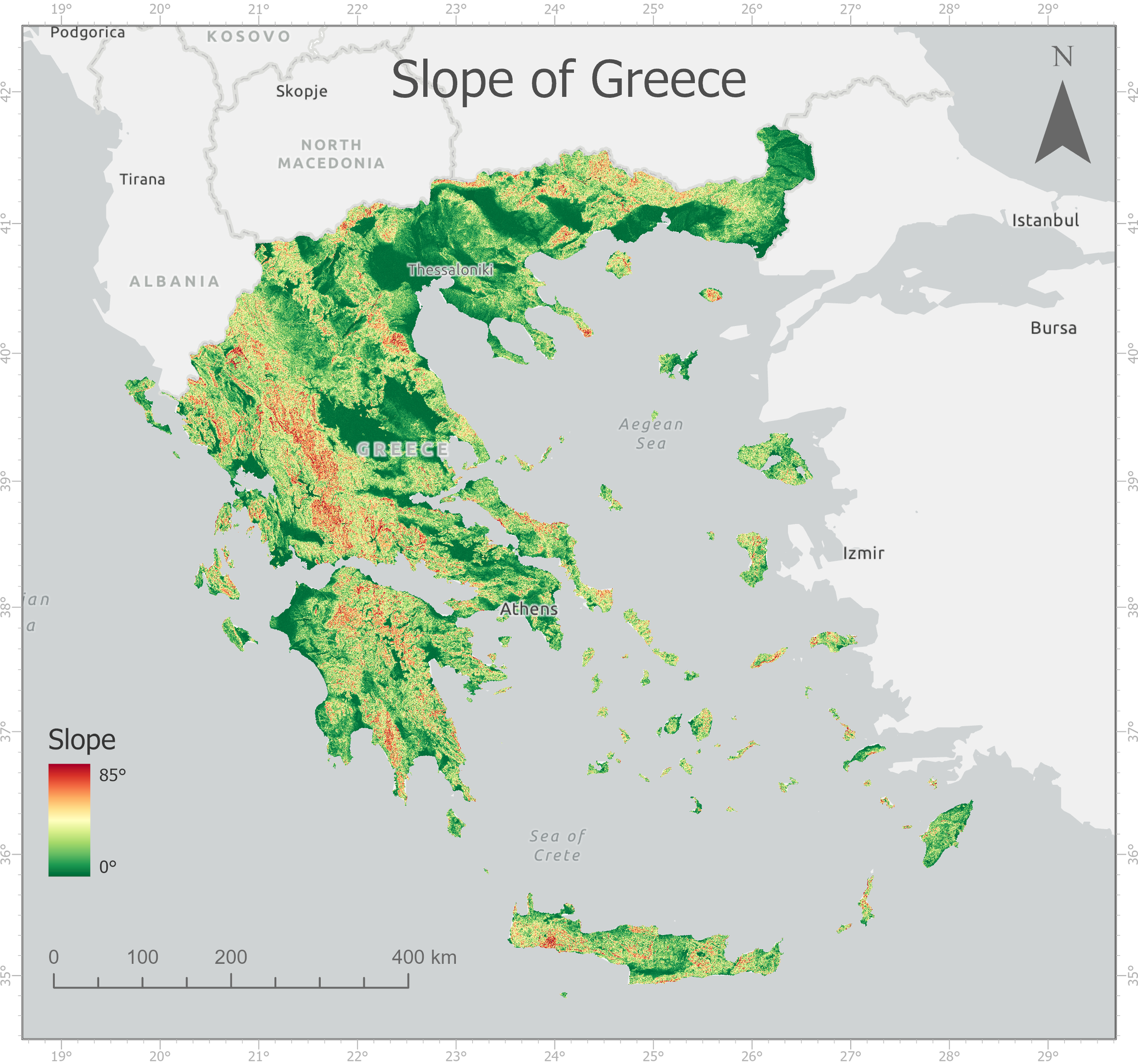

Supplement: S6 Fig — (TIF) [file pone.0339998.s006.tif]

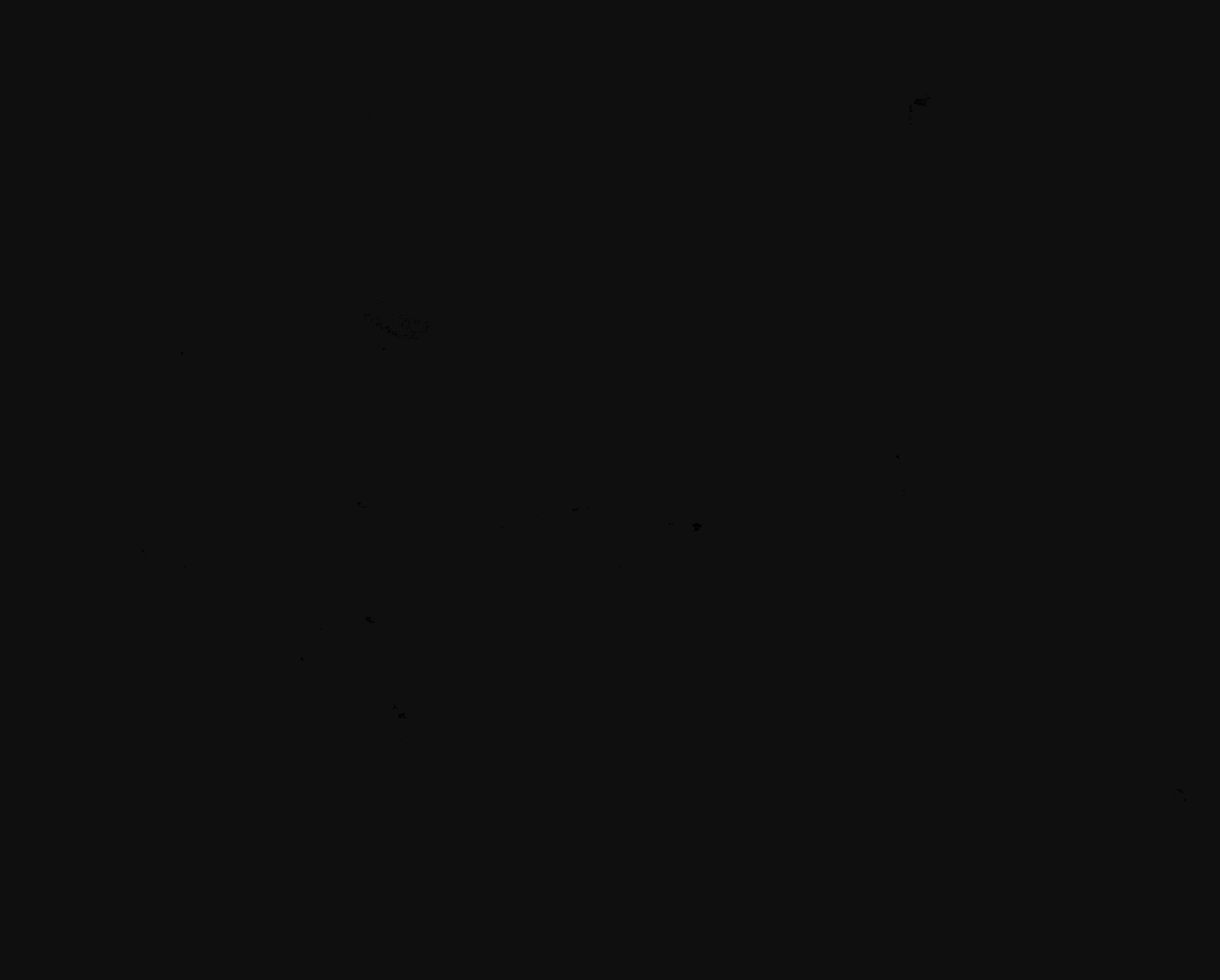

Supplement: S3 File — (ZIP) [file pone.0339998.s009.zip › Palenova_Prioritisation_Minimal_data_set/overlay_just_priority.tif]

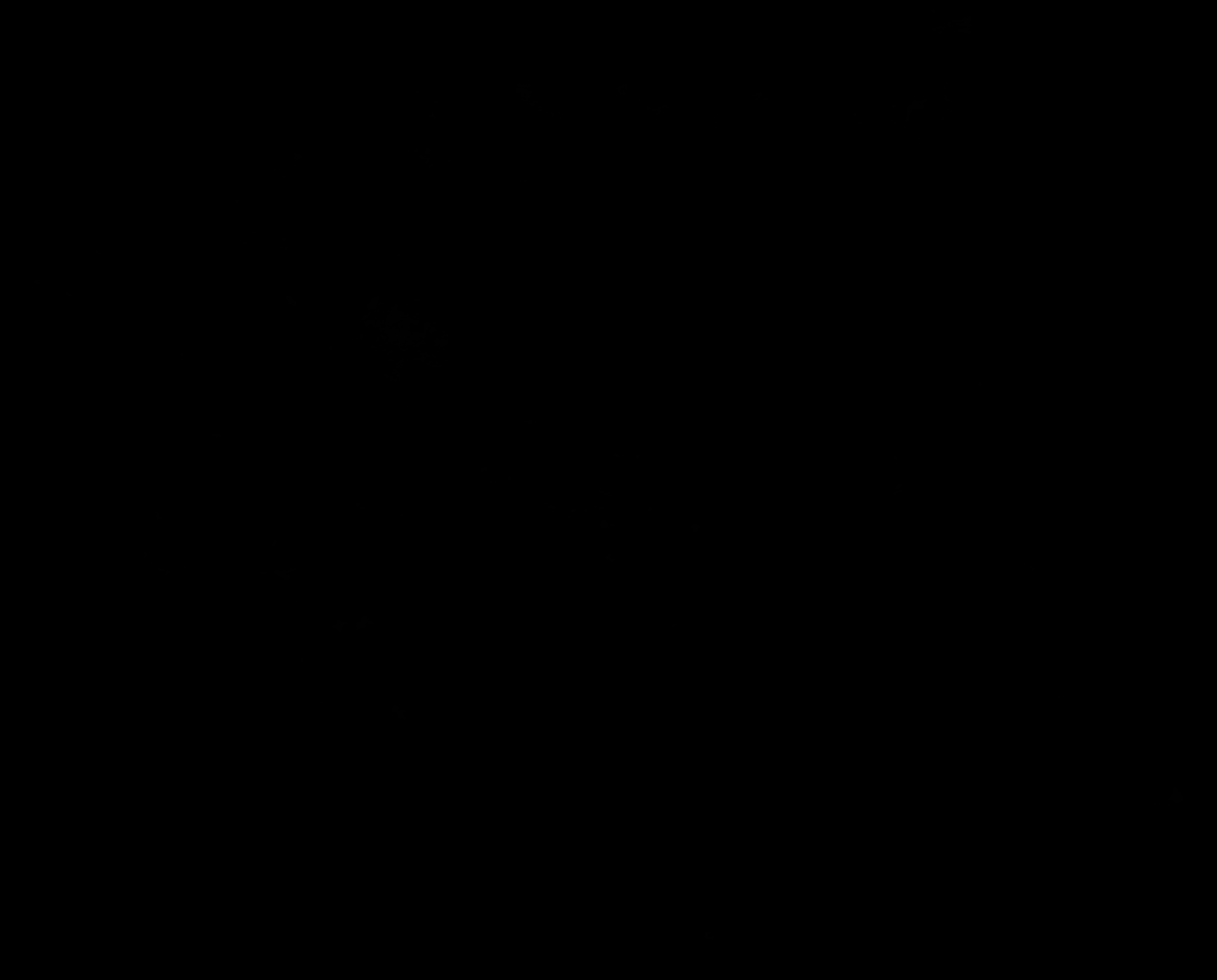

Supplement: S3 File — (ZIP) [file pone.0339998.s009.zip › Palenova_Prioritisation_Minimal_data_set/repetitive_burns_reclas.tif]

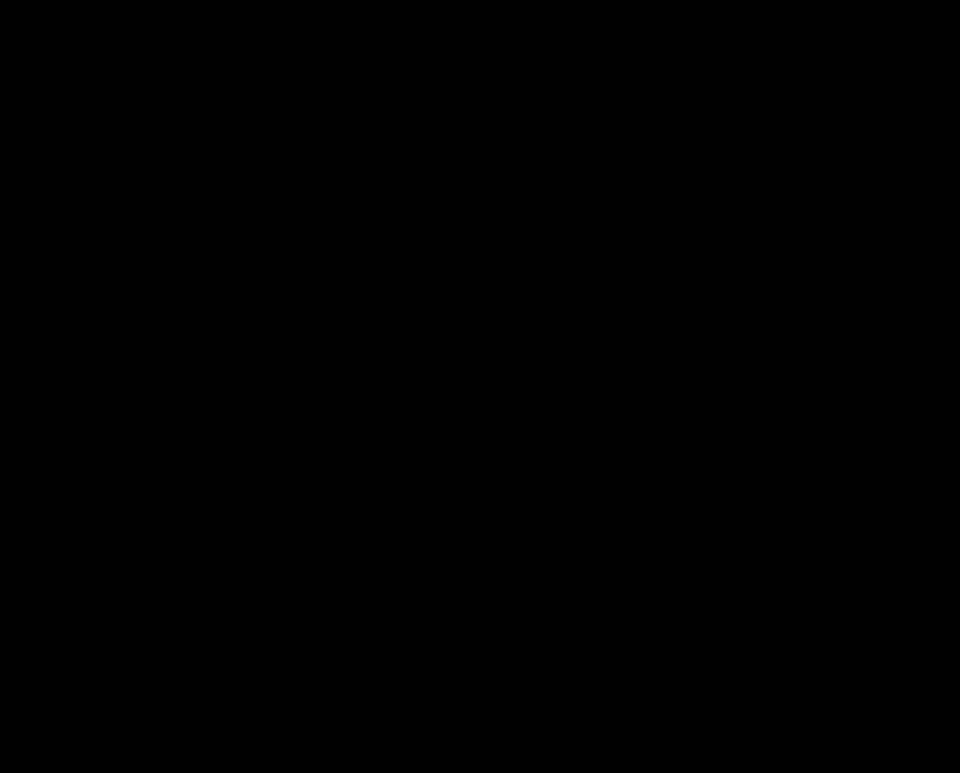

Supplement: S3 File — (ZIP) [file pone.0339998.s009.zip › Palenova_Prioritisation_Minimal_data_set/repetitive_burns_reclas.tif.ovr]
