# Supplementary material for: Prioritizing areas for post-fire restoration in Greece using mixed-methods spatial analysis
Source: PLoS One. 2026 Jan 12;21(1):e0339998. doi: 10.1371/journal.pone.0339998 (PMC12795358; doi:10.1371/journal.pone.0339998)
Supplement: S1 File — Appendices 1–5. (DOCX) [file pone.0339998.s007.docx]

## Prioritizing Areas for Post-Fire Restoration in Greece Using Mixed-Methods Spatial Analysis

Elena Palenova^^[[1]](#footnote-1)^^, Sander Veraverbeke^^[[2]](#footnote-2)^,^[[3]](#footnote-3)^^, Igor Drobyshev^^[[4]](#footnote-4)^^, Themistoklis Kontos^^[[5]](#footnote-5)^^, Karin Ebert^1^

Corresponding author

Email: [elena.palenova@sh.se](mailto:elena.palenova@sh.se) (EP)

# Supporting information

## Appendix

### Appendix 1. Selected interview questions about views on post-fire management in Greece

- Regarding wildfire management in Greece, what is the main goal or set of goals nowadays? What needs to be reached?
- How do you see the situation with wildfires, and, specifically, restoration after wildfires in Greece now?
- What are the main problems related to vegetation restoration you can highlight?
- What are the causes/main sources of problems with vegetation restoration after wildfires?
- Do you know any working systems of restoration after wildfires in Greece?
- What influenced the current state of the issue of vegetation recovery after wildfires in Greece?
- What should be done on our way to the goals? What intermediate strategies and objectives to implement?
- What are the actions step by step as you see it?
- Which wildfire management approaches can better help with restoration most cost-effectively?
- What areas should be prioritised when it comes to making restoration efforts after wildfires?
- What are the most important activities that need to be done on land after wildfires?
- What increases restoration complexity and costs? What can decrease them?
- What should be done to address future fire consequences/restoration practices?

### Appendix 2. Latest Annual Burn Dates for Greece with MODIS Data, in GEE in JavaScript

// Import shapefile for Greece

var greece = ee.FeatureCollection(geometry);

// Function to calculate the latest burn date for a given year and return the result as an image

function calculateBurnDateForYear(year) {

// Define the start and end dates for the year

var startDate = ee.Date.fromYMD(year, 1, 1);

var endDate = ee.Date.fromYMD(year, 12, 31);

// Load the MODIS burned area dataset for the given year

var dataset = ee.ImageCollection('MODIS/061/MCD64A1')

.filter(ee.Filter.date(startDate, endDate));

// Select the 'BurnDate' band

var burnedArea = dataset.select('BurnDate');

// Clip the dataset to Greece

var burnedAreaGreece = burnedArea.map(function(image) {

return image.clip(greece);

});

// Get the latest burn date for each pixel by taking the max value over the time period

var burnedAreaLatest = burnedAreaGreece.max();

// Return the latest burn date image

return burnedAreaLatest.rename('latest_burn_date');

}

// Loop through each year from 2000 to 2024 and export each year's latest burn date as a GeoTIFF

for (var year = 2000; year <= 2024; year++) {

var burnedDateImage = calculateBurnDateForYear(year);

// Export the latest burn date image to Google Drive as a GeoTIFF

Export.image.toDrive({

image: burnedDateImage,

description: 'LatestBurnDate_Greece_' + year,

scale: 500, // Resolution of MODIS

region: greece.geometry(),

fileFormat: 'GeoTIFF',

folder: 'MODIS_Appendix_1',

fileNamePrefix: 'latest_burn_date_greece_' + year,

maxPixels: 1e13

});

}

### Appendix 3. Raster Algebra and Visualization of Fire Occurrence Using Python in Google Colab

import os

import rasterio

import numpy as np

folder_path = '/content/drive/MyDrive/fires/MODIS_061_MCD64A1/'

# List all .tif files in the folder

tif_files = [os.path.join(folder_path, f) for f in os.listdir(folder_path) if f.endswith(".tif")]

# Read the first file to get dimensions and metadata

with rasterio.open(tif_files[0]) as src:

profile = src.profile

raster_shape = src.shape # (height, width)

# Initialize sum array and mask

sum_raster = np.zeros(raster_shape, dtype=np.float32)

burned_mask = np.zeros(raster_shape, dtype=np.uint8)

# Process each raster file

for tif_file in tif_files:

with rasterio.open(tif_file) as src:

data = src.read(1) # Read first band

sum_raster += data # Sum rasters

burned_mask[data > 0] = 1 # Mask where burned areas occurred at least once

# Define output file paths

sum_output_path = os.path.join("/content/drive/MyDrive/fires/MODIS_061_MCD64A1/summed_raster.tif")

mask_output_path = os.path.join("/content/drive/MyDrive/fires/MODIS_061_MCD64A1/burned_mask.tif")

# Update metadata

profile.update(dtype=rasterio.float32, count=1)

# Save the summed raster

with rasterio.open(sum_output_path, 'w', **profile) as dst:

dst.write(sum_raster, 1)

# Save the burned area mask (binary output)

profile.update(dtype=rasterio.uint8)

with rasterio.open(mask_output_path, 'w', **profile) as dst:

dst.write(burned_mask, 1)

# Plot rasters

plt.figure(figsize=(10, 5))

plt.subplot(1, 2, 1)

plt.imshow(sum_raster, cmap='hot')

plt.colorbar()

plt.title("Summed Raster")

plt.subplot(1, 2, 2)

plt.imshow(burned_mask, cmap='gray')

plt.title("Burned Area Mask")

plt.show()

# Calculate the area with MODIS MCD64A1 spatial resolution

pixel_size_km2 = (500 * 500) / 1_000_000 # Convert to km²

burned_pixel_count = np.sum(burned_mask == 1)

total_burned_area_km2 = burned_pixel_count * pixel_size_km2

print(f"Total Burned Area: {total_burned_area_km2:.2f} km²")

### Appendix 4. Raster Algebra and Visualisation for Repetitively Burned Areas Using Python in Google Colab

import os

import rasterio

import numpy as np

import matplotlib.pyplot as plt

# Directory where the rasters are stored

raster_dir = '/content/drive/MyDrive/fires/MODIS_061_MCD64A1/'

# List all raster files

raster_files = [os.path.join(raster_dir, f) for f in os.listdir(raster_dir) if f.endswith('.tif')]

# Read the rasters and stack them

rasters = []

for raster_file in raster_files:

with rasterio.open(raster_file) as src:

rasters.append(src.read(1))

# Stack the rasters into a 3D numpy array

stacked_rasters = np.stack(rasters, axis=0)

# Sum all rasters across the stack

burned_areas_sum = np.sum(stacked_rasters, axis=0)

# Create a mask where burned areas occurred more than once

burned_more_than_once = burned_areas_sum > 1

# Define the path for the output raster

output_raster = '/content/drive/MyDrive/fires/MODIS_061_MCD64A1/burned_more_than_once.tif'

# Copy metadata from one of the original rasters

with rasterio.open(raster_files[0]) as src:

meta = src.meta.copy()

# Update metadata to reflect the number of layers (single layer output)

meta.update(dtype=rasterio.uint8, count=1)

# Write the output raster

with rasterio.open(output_raster, 'w', **meta) as dst:

dst.write(burned_more_than_once.astype(rasterio.uint8), 1)

# Plot the areas that burned more than once

plt.imshow(burned_more_than_once, cmap='Reds')

plt.title('Areas Burned More Than Once')

plt.colorbar()

plt.show()

# Calculate area with MODIS MCD64A1 spatial resolution

pixel_size_km2 = (500 * 500) / 1_000_000

burned_pixel_count = np.sum(burned_more_than_once == 1)

total_burned_area_km2 = burned_pixel_count * pixel_size_km2

print(f"Total Burned Area: {total_burned_area_km2:.2f} km²")

### Appendix 5. Slope of Greece.


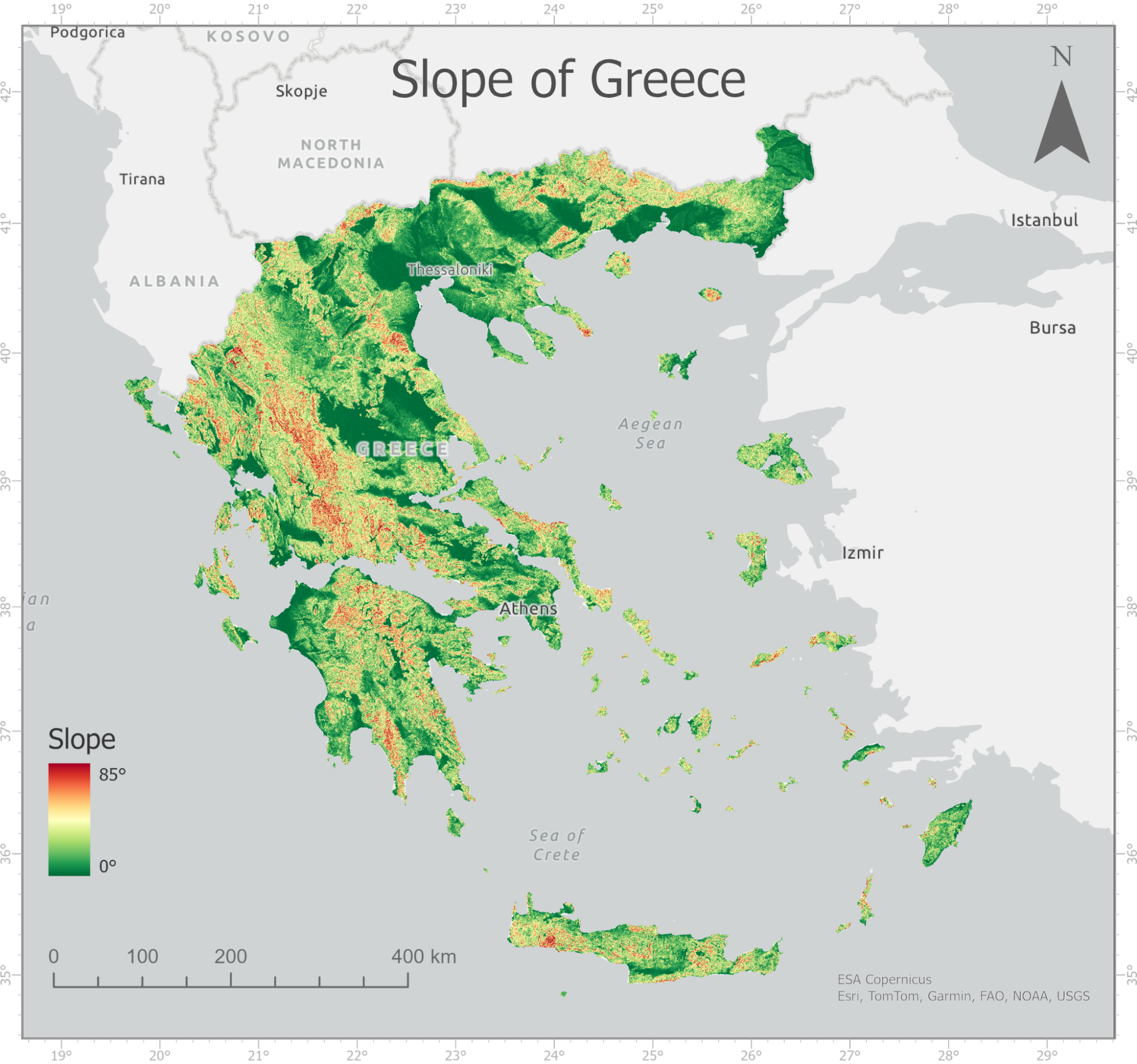


1. Department of Natural Sciences, Technology and Environmental Studies, Södertörn University, Stockholm 14189, Sweden [↑](#footnote-ref-1)
2. Faculty of Science, Vrije Universiteit Amsterdam, 1081 HV Amsterdam, Netherlands [↑](#footnote-ref-2)
3. School of Environmental Sciences, University of East Anglia, NR4 7TJ Norwich, United Kingdom [↑](#footnote-ref-3)
4. Department of Southern Swedish Forest Research Centre, Swedish University of Agricultural Sciences, Alnarp 23456, Sweden [↑](#footnote-ref-4)
5. Department of Environment, University of the Aegean, Mitilini 81100, Greece [↑](#footnote-ref-5)
