# Supplementary material for: Prioritizing areas for post-fire restoration in Greece using mixed-methods spatial analysis
Source: PLoS One. 2026 Jan 12;21(1):e0339998. doi: 10.1371/journal.pone.0339998 (PMC12795358; doi:10.1371/journal.pone.0339998)
Supplement: Attachment — Submitted filename: Response to Reviewers v2.docx [file pone.0339998.s010.docx]

PLOS ONE

PONE-D-25-26522

Prioritizing Areas for Post-Fire Restoration in Greece Using Mixed-Methods Spatial Analysis

**Response to Reviewers**

by Elena Palenova

**Regarding previous commets by Ronalyn M. Ramos:**

1. I uploaded data received through the GIS analysis as the Supporting Information file, zip-file.

2. I removed figures from my manuscript. You have already asked me to remove it during the first editing, then asked to put back, and now asked to remove again. I will do anything according to guidelines, just let me know.

3. All datasets used in figures are publicly available and can be used in research with proper referencing, which meets PLOS ONE requirements for CC BY 4.0 license. No copyrighted base maps or proprietary data (e.g., Google Maps, Copernicus satellite imagery under restrictive terms) were used.

- MCD64A1.061 MODIS Burned Area Monthly Global 500 m by NASA LP DAAC; license is public domain, use is unrestricted, including for redistribution and publication.

- Copernicus Digital Elevation Model (DEM) by DLR & Airbus (2010–2018); license is Copernicus Free and Open Data License, use is free for commercial and non-commercial purposes with proper citation.

- World Database on Protected Areas (WDPA) by UNEP-WCMC; license is free to use for non-commercial purposes (including academic research), use is permitted with proper attribution, with no prior permission required for academic use.

- Natural Earth from naturalearthdata.com; license is public domain; use is free for any purpose—personal, academic, or commercial—with no restrictions on modification, redistribution, or publication.

Attribution is provided within the manuscript, please let me know if extra references on the figures, on the maps themselves should be removed! I will do it.

**Regarding Additional Editor Comments:**

1. I included continuous line numbers in my submission

2. I included figures within the text in Word format

**Regarding Journal Requirements:**

1. I changed the text formatting based on body formatting guidelines and title, author, affiliation formatting guidelines, added supporting information and changed names of attached files. Let me know if I am missing something and I will make all changes!
2. I included the questionnaire on inclusivity in global research as supporting file and added information to the Methods section.
3. Code sharing information is given in Declarations - Data Availability Statement. In “Recommendations for sharing code” it says it is “encouraged” to share the code in repository with DOI and licenses etc., but not obliged, so I would prefer to have a code as it is now, in Appendices, and in Data Availability Statement of the manuscript mentioning how the code can be accessed. If it is an obligation, please let me know and I will follow all necessary directions!
4. Details regarding paritcipant consent were added both to Methods section and “Ethics Statement” field of the submission form in the previous review.
5. The submission follows the LaTeX format, however, it is a bit confusing regarding changing the Word format to PDF and recommendations such as "do not include figures in your PDF; they should be uploaded as separate files", because they contradict instructions of submitting Word file with tracked changed and include Figures there. Also, there are some differences in LaTeX format and Manuscript body formatting guidelines I followed, for example, Supporting information being before or after References and many other small differences. Maybe I am mixing up things, but please let me know which directions to follow, and I will do everything that is needed!
6. Data used in Figures 1-5 and Appendix 5 are from open sources/in public domain that can be used under the Creative Commons Attribution License (CC BY 4.0). It includes:

- Digital elevation model (DEM) from Copernicus

- MCD64A1.061 MODIS Burned Area Monthly Global 500m data product from NASA

- Boundaries of protected areas from the World Database on Protected Areas

- Administrative boundaries from the Natural Earth database

All used data is available under CC BY 4.0 license and properly cited in the manuscript. Maps were created using ArcGIS Pro software licensed through my university. Google Earth Engine was used only as a tool of retrieving MODIS (NASA) data, but not for creating figures for the article. Let me know if I need to edit maps, remove background copyrights available there from the basemap, etc.

7. All personal information is removed already, submitted file is anonymized.
8. I included captions for my Supporting Information files at the end of my manuscript. In the text body, figures are named as "Fig 1. Fire Frequency in Greece (2001-2023).", in Supporting Information at the end, they are named "S1 Fig. Fire Frequency in Greece (2001-2023)." I hope it is correct way to do it, but let me know if no, and I will edit and fix everything!

**Regarding other comments:**

I uploaded files to <https://pacev2.apexcovantage.com/> and replaced original source files with PACE generated files, both in Word and as attached supporting files.
