# Supplementary material for: Prioritizing areas for post-fire restoration in Greece using mixed-methods spatial analysis
Source: PLoS One. 2026 Jan 12;21(1):e0339998. doi: 10.1371/journal.pone.0339998 (PMC12795358; doi:10.1371/journal.pone.0339998)
Supplement: Attachment — Submitted filename: Response to Reviewers.docx [file pone.0339998.s011.docx]

Thank you for your reviews! Answers to the reviewers’ suggestions are highlighted below in blue

**Reviewer 1:**

This manuscript addresses a highly relevant and timely topic, namely the prioritization of areas for post-fire restoration in Greece using a mixed-methods approach that integrates stakeholder perspectives with GIS and remote sensing analysis. The study is well-structured, uses openly available datasets, and offers practical insights that could inform restoration planning and policy. Its novelty lies in combining expert interviews with geospatial analysis, which provides a socio-ecological dimension often missing from purely technical frameworks.

Thank you!

However, the manuscript would benefit from further strengthening in the Introduction, Methods, and Discussion to better situate the work in the existing literature and to clarify its methodological contributions.

I added more research literature in Introduction and added additional information accordingly to Methods in Discussion. Hopefully now it is placing the research better in the context of existing research and clarity methodological contributions.

The Introduction section needs to be enhanced. At present, it provides a good overview of wildfire impacts and post-fire management in Greece but lacks sufficient contextualization of the role of GIS and remote sensing in prioritization frameworks. I recommend adding a dedicated paragraph on the use of GIS-based approaches for restoration prioritization, referencing relevant international and Mediterranean studies. This will help position your mixed-methods approach in relation to existing geospatial methodologies and highlight the novelty of integrating stakeholder input.

Some suggested references you could include are:
• Dosis, Stefanos, George P. Petropoulos, and Kleomenis Kalogeropoulos. "A geospatial approach to identify and evaluate ecological restoration sites in post-fire landscapes." Land 12.12 (2023): 2183.
• Prodromou, M.; Gitas, I.; Mettas, C.; Tzouvaras, M.; Themistocleous, K.; Konstantinidis, A.; Pamboris, A.; Hadjimitsis, D. Remote-Sensing-Based Prioritization of Post-Fire Restoration Actions in Mediterranean Ecosystems: A Case Study in Cyprus. Remote Sens. 2025, 17, 1269.

I added two paraghaphs with review of literature regarding using GIS and Remote Sensing techniques for restoration prioritisation, considering after-disaster restoration and specifically restoration after wildfires, both international and Mediterranean contexts, to highlight the novelty of the research and filling the existing gaps, and also blended Introduction and Discussion with new sources. Suggested references are reviewed and added as well.

The current subtitle “Mixed methods” could be revised to a more professional and descriptive term. I recommend using a title such as “Methodological Framework”, “Study Design and Analytical Methods”, or “Methodological Approach”. This will better reflect the integration of qualitative (stakeholder interviews) and quantitative (GIS/RS analysis) components and improve the overall readability of the manuscript.

Thank you for this suggestion, I changed the title to the recommended title

Discussion – Methodological Remarks
In lines 513–525, where you discuss the methodological remarks, I recommend strengthening the comparison with other studies that have addressed the selection of criteria for post-fire restoration prioritization. This would situate your work more firmly within the existing literature and highlight how your criteria (repetitive burns, slope, protected areas) align with or differ from approaches in previous research.

In “Methodological Remarks”, I added the paragraph in the beginning and blended research into the chapter body with the comparison with other research on criterias for post fire restoration using the literature from Introduction and adding several additional studies, therefore highlighted how the reseach fit into existing knowledge and showing the methodological novelty

Additionally, as a suggestion for future work, it would be valuable to mention the potential for validation of your framework, for example by comparing the identified priority areas with outcomes of existing restoration actions in Greece or in other Mediterranean fire events. This would enhance the practical relevance and robustness of the proposed methodology.

Added paragraph at the end of Discussion section on possibilities for the framework validation. I named it “Framework validation and data gaps.” It is a good and potentially practically useful suggestion, thank you very much!

**Reviewer 2:**

The manuscript addresses an important topic and presents valuable data on prioritizing areas for post-fire restoration in Greece using a mixed-methods spatial approach. However, several aspects require substantive revision before the manuscript is suitable for publication.

Thank you!

Terminology consistency: The manuscript currently alternates between “wildfire,” “fire,” and “burns.” It would improve clarity and readability to use a consistent term throughout, e.g., “wildfire” for natural fires, and avoid switching to “burns.”

Changed “burnes” into fires! Good suggestion to avoid “burns”

Abstract
I suggest not including the abbreviations GIS and RS in parentheses in the abstract. It is sufficient to use the full terms there and then introduce the abbreviations later in the main text, for example in the Materials and Methods section, as currently done.

I did it because it was a recommendation from my co-author, but I also prefer not to include abbreviations in Abstract. So, removed!

Introduction
I noticed repeated citations of the same source – article no. 1 is cited in lines 41, 43, 58, and 61. It would be advisable to find additional sources for these statements, or to include more relevant references, especially considering the length and importance of these sections in the Introduction.

Removed two mentioning of article 1 and replaced them with different relevant papers (see references 2,3,11)

In lines 54–58, various damages caused by fires are mentioned, but it would be appropriate to also include damages to forests, since forests are frequently mentioned later in the text, as well as reforestation.

Mentioned forests

Use the term wildfire whenever referring to a forest fire. For example, in line 62 (both occurrences) and line 108. Of course, the terms fire severity and post-fire restoration are correctly used and do not require “wildfire.”

Replaced, left “fire-prone weather” and “fire season” because these are stable terms

The sentence in lines 65–66 contains too many instances of “and.” I suggest rephrasing it, for example:
“The fires caused extensive damage to infrastructure, properties, and natural reserves, impacting biodiversity and local economies.”

Rephrased

In lines 68–69, it is stated that vegetation recovery generally occurs without intervention, with only a single citation (no. 11). I do not entirely agree with this, as replanting is frequently practiced (also mentioned in line 80 regarding reforestation in Greece). Either this statement should be supported by additional references, or the sentence should be reformulated.

Updated with one more article, more up-to-date one

From lines 88 to 99, I recommend standardizing the names of ministries and checking capitalization to ensure consistency.

Capitalised, standardised the names

In line 96, the term emergency stabilization should be clarified (stabilization of what?).

Added “soil”

In line 100, “post-fire erosion risk” should be specified more clearly, e.g., soil erosion.

Added “soil”

Material and methods
The manuscript uses the section title “Material and methods,” which is unusual. In last published PLOS ONE articles, the section is simply labelled “Methods,” although the journal’s submission guidelines recommend “Materials and Methods.” Consider adjusting the section title to align with typical journal usage.

Academic Editor disagreed with this suggestion, so I guess I will leave as it is now!

Mixed methods section: The “Mixed methods” section reads more like part of the Introduction. In the Materials and Methods section, the focus should be on providing a detailed description of the procedures, data, analysis steps, selection of stakeholders, software used, and other methodological details. A graphical representation of the workflow would also be very helpful, as it would give the reader a clear overview of the process from stakeholder interviews to GIS analysis and the prioritization of areas. The paragraphs currently in lines 141–157 could be retained for context but should be moved to the Introduction, while the Materials and Methods should clearly outline the actual steps taken in the study.

Removed most part of it to the Introduction

Interviews section: I suggest reorganizing this section. First, it would be helpful to present the paragraph describing what was done in the study (currently lines 181–187), so that the reader clearly understands the procedures, topics, duration, and recording of the interviews. Only after that should the manuscript provide details on who was interviewed, including affiliations and the table currently in lines 161–178.
Rationale: If the reader first sees what was done (number of interviews, topics, duration, recording), they can better understand the context for the subsequent information on who was interviewed. This order allows readers to first grasp the methods and then see the additional details about the respondents, improving clarity and flow of the text.

Reorganised, restructured, good direction!

Selecting recurrent criteria: This section clearly presents the criteria identified from the interviews, which is very helpful. I recommend adding a brief explanation of why only criteria mentioned by at least two respondents were included and how this threshold was determined.

Added bried explanation

Geospatial data: In this paragraph, it would improve clarity if the order of the data description matched the later table and detailed source section. For example, start with historical burned area data, then describe the DEM and slope data, and finally the protected areas. This ensures consistency and helps the reader follow the workflow from data selection to analysis. Additionally, it would be helpful to specify the time period covered by the historical burned area data, so that readers clearly understand the temporal scope of the dataset.

Restructured and added!

Abbreviation usage: Consider using the abbreviation GEE for Google Earth Engine in line 233 for consistency and brevity.

Done

Results
Results clarity: The Results section clearly presents the outcomes of the analysis, but the description could be improved by consistently using terminology (e.g., “wildfire” instead of “fire” or “burned”) and by ensuring that figures and tables are referenced in a logical order matching the text.

Changed to “wildfires”

It might improve readability if the presentation of results followed a consistent order: start with historical burned area data, then repetitive fires, slope analysis, and finally protected areas and overlay results. Additionally, providing percentages alongside absolute areas (as done in some cases) for all metrics would help readers quickly understand the relative scale of each criterion.

The order is exactly as you mention – historical data, repeated wildfires, slope, protected areas

Discussion
Selected areas, Restoration measures, and Additional insights: Although these sections are labelled as Discussion, they mostly read as extended descriptions of results, case-specific examples, and practical recommendations, rather than analytical interpretation. They provide detailed accounts of selected areas, post-fire actions, community involvement, staffing, use of GIS, and funding challenges. In a typical Discussion, one would expect:
• comparison of findings with previous studies (e.g., “Our results are consistent with XY et al. (number)…”,
• interpretation of observed patterns and their ecological, social, or policy implications,
• acknowledgement of study limitations (currently addressed later in “Methodological remarks”), and
• broader implications for management, policy, or future research.
As currently written, the sections resemble Results or applied Recommendations. Reframing them to focus on analysis, interpretation, and synthesis would strengthen the manuscript and make it more aligned with standard scientific discussion structure.

To address this comment, I expanded the research literature in the Introduction and the Discussion and added corresponding methodological details to the Discussion. I hope this better situates the study within the context of existing research and clarifies its methodological contributions.

Methodological remarks section: This is more like discussion, as it evaluates the approach, its strengths, limitations, and possibilities for extension. However, to constitute a discussion of manuscript, it would be important to compare the findings with previous studies, so that it is clear what is novel and what confirms or differs from existing knowledge.
I recommend adding statements such as:
• “Our findings are in line with XY et al. (number linked to references) who also highlighted the role of double-burned areas on steep slopes for restoration priorities.”
• “Unlike previous studies (AB et al., number linked to references), we integrated qualitative insights from stakeholders with geospatial analysis, providing a more nuanced perspective on post-fire management.”
• “The observed patterns of natural recovery correspond with findings reported by CD et al. (number linked to references) in Mediterranean ecosystems, emphasizing the importance of considering local socio-ecological constraints.”
This way, the section would not only evaluate the methodology and results but also situate the findings within the context of existing literature, which is exactly what a typical discussion should provide.

This is a good direction which was also addressed by the first reviewer. I added a new paragraph in the beginning of the section where I compared our findings with existing studies on criteria for post-fire restoration. This includes new reviewed and added literature from the Introduction as well as several additional sources. I guess this is how I clarified how the research fits within the current knowledge and highlighted the methodological novelty. Also, I added a concluding paragraph to the Discussion section named **“Framework validation and data gaps”** with potential approaches for validating the framework.

Conclusions:
The conclusion effectively summarizes the main findings of the study and emphasizes the practical applicability of the mixed-methods approach for prioritizing post-fire areas.

Thank you!


Overall, the manuscript has strong potential, but substantive revisions—especially in the Discussion and Introduction—are necessary to fully meet the standards of analytical interpretation and contextualization in the literature.

Done, hopefully it is good now!
